# New Hyaluronic Acid from Plant Origin to Improve Joint Protection—An In Vitro Study

**DOI:** 10.3390/ijms23158114

**Published:** 2022-07-23

**Authors:** Rebecca Galla, Sara Ruga, Silvio Aprile, Sara Ferrari, Arianna Brovero, Giorgio Grosa, Claudio Molinari, Francesca Uberti

**Affiliations:** 1 Laboratory of Physiology, Department of Translational Medicine, University of Piemonte Orientale, Via Solaroli 17, 28100 Novara, Italy; rebecca.galla@uniupo.it (R.G.); sara.ruga@uniupo.it (S.R.); sara.ferrari@uniupo.it (S.F.); arianna.brovero@gmail.com (A.B.); 2 Dipartimento di Scienze del Farmaco, Università del Piemonte Orientale, Largo Donegani 2, 28100 Novara, Italy; silvio.aprile@uniupo.it (S.A.); giorgio.grosa@uniupo.it (G.G.); 3 Dipartimento per lo Sviluppo Sostenibile e la Transizione Ecologica, University of Piemonte Orientale, 13100 Vercelli, Italy; claudio.molinari@uniupo.it

**Keywords:** osteoarthritis, cartilage inflammation, tissue degradation, high molecular weight hyaluronic acid, intestinal absorption, chondrocytes

## Abstract

Background: In recent decades, hyaluronic acid (HA) has attracted great attention as a new treatment option for osteoarthritis. Classical therapies are not able to stop the cartilage degeneration process nor do they favor tissue repair. Nowadays, it is accepted that high molecular weight HA can reduce inflammation by promoting tissue regeneration; therefore, the aim of this study was to verify the efficacy of a new high molecular weight HA of plant origin (called GreenIuronic^®^) in maintaining joint homeostasis and preventing the harmful processes of osteoarthritis. Methods: The bioavailability of GreenIuronic^®^ was investigated in a 3D intestinal barrier model that mimics human oral intake while excluding damage to the intestinal barrier. Furthermore, the chemical significance and biological properties of GreenIuronic^®^ were investigated in conditions that simulate osteoarthritis. Results: Our data demonstrated that GreenIuronic^®^ crosses the intestinal barrier without side effects as it has a chemical–biological profile, which could be responsible for many specific chondrocyte functions. Furthermore, in the osteoarthritis model, GreenIuronic^®^ can modulate the molecular mechanism responsible for preventing and restoring the degradation of cartilage. Conclusion: According to our results, this new form of HA appears to be well absorbed and distributed to chondrocytes, preserving their biological activities. Therefore, the oral administration of GreenIuronic^®^ in humans can be considered a valid strategy to obtain beneficial therapeutic effects during osteoarthritis.

## 1. Introduction

Osteoarthritis (OA) is a slow progressive joint disorder that causes several disabilities in the adult population [1]. For a long time, OA was regarded as progressive wear of the joint cartilage alone. However, recent research has shown that it is an inflammatory disease of the entire synovial joint, which includes not only the mechanical degeneration of the articular cartilage, but also the concomitant structural and functional change of the entire joint, including the synovium, meniscus, periarticular ligaments, and subchondral bone [2]. An important role in infrapatellar fat pad inflammation and fibrosis has also recently been discovered [3]. Today, the treatment modalities for OA include non-pharmacological (e.g., physiotherapy), pharmacological (e.g., steroidal and nonsteroidal anti-inflammatory drugs), or intra-articular (e.g., injection of hyaluronic acid) therapies [4,5]. These classical therapies can reverse the symptoms only in a small number of cases, but they do not stop the degeneration process of the cartilage or promote the repair of the tissue. Therefore, the development of new therapies is a primary goal, preferably hypothesizing the oral administration, which remains the preferred route for drug delivery due to its low invasiveness, high efficiency, and better patient compliance [6]. Obviously, in this case, the bioaccessibility and bioavailability of orally administered compounds need to be investigated in a preclinical model in order to evaluate the ability to cross the intestinal barrier after oral administration. During the last decades, hyaluronic acid (HA) has attracted great attention as a new treatment option for knee OA pain [7,8,9]. HA is a natural polymer belonging to the glycosaminoglycan heteropolysaccharides family (GAGs), but unlike these molecules, it is not sulfated and it is not synthesized by Golgi enzymes [10]. In addition, the native form appears as a very long polymer, called high-molecular weight HA (HMWHA) [10]. Therefore, the native HA consists of 2000–25,000 disaccharide units, corresponding to 10^6^–10^7^ Da molecular weight; for that, a long chain contains more than 10,000 units, which is ~4000 kDa [9,11]. In the biological systems, HMWHA (also called native HA) is degraded into small fragments named low molecular weight HA (LMWHA) corresponding to different molecular weights; in particular, HMWHA has >1 to 10 MDa; intermediate HA has >100 to 1000 kDa; and LMWHA has the molecular weight between 1 and 10 kDa [9,12]. Several studies reported that the structural and biological properties of HA within medical, pharmaceutical, and cosmetic applications analyzing the role of HA in inflammation and tissue regeneration are related to its specific molecular weight [13,14]. Applications of HA depend on its biological effects on cell differentiation and proliferation, and on its ability to lubricate, hydrate, and interact with various receptors present on the cell surface. It is this interaction that facilitates the exact delivery of drugs, facilitating their internalization in target sites. The safety, tolerability, and efficacy of HA-based formulations for the treatment of various types of joint diseases have been validated in several studies [10,15]. It is widely accepted that exogenous hyaluronic acid is incorporated into articular cartilage where it may have a direct biological effect on chondrocytes to improve joint lubrication as well described by clinical studies. The concept of viscosupplementation is based upon the hypothesis that HA administration could improve the rheological properties of joints, and promote the endogenous synthesis of HMWHA and possibly more functional HA, thereby improving mobility, and articular function, and decreasing pain. The growing use of HA in medical practice can be explained by its effectiveness and versatility as well as its favorable safety profile [16]. Nowadays, sodium HA seems to be the best choice available on market, since it exerts an analgesic effect by blocking pain receptors in synovial tissues and holding endogenous pain substances in its molecule [17]. However, it can be suggested that the characteristic steric configurations of HMWHA are necessary for the manifestation of the analgesic effect, indicating a possible clinical application of all HA fragments [17]. Indeed, HA is a Food and Drug Administration (FDA)-approved treatment for inflammatory conditions, including those affecting the joints, and is also acknowledged in Europe for its beneficial properties due to the therapeutic potential caused by native HA, but without toxicity [9]. In particular, in vitro studies demonstrate that only native HA exerts an inhibitory effect on interleukin (IL)-1βstimulated prostaglandin E2 (PGE2) production in inflammation-damaged bovine cartilage. This supports the hypothesis that administration of HMWHA may be an important new strategy to restore proteoglycan content leading to a new cartilage protective strategy [9,18]. Synovial inflammation and structural and molecular changes in the joint system should be the target of OA therapies. For this reason, thanks to its viscoelastic properties, HA therapy has been proposed for OA [19]. Much research has been conducted on HA combined with anti-inflammatory drugs, both in clinical trials and in vivo and in vitro studies, and the published data indicate that, with a good level of significance, intra-articular injection of HA combined with anti-inflammatory drugs can potentially relieve pain in OA knee patients [20]. In the last years, symptomatic slow-acting drugs for OA have been vastly studied and many studies have focused the attention on HA, or chondroitin sulfate (CS) combined with nonsteroidal anti-inflammatory drugs (NSAIDs) to limit the related adverse events in the gastrointestinal tract, kidney, and cardiovascular system [19]. Finally, to reduce the strong adverse effects due to drugs, HA may be combined with several agents including lactose-modified chitosan and cyclodextrin to improve chondroprotection and to stimulate cartilage growth reducing inflammation [21,22]. Additional studies reported similar beneficial effects of HMWHA in OA and in other inflammatory conditions [23,24]. The main purpose induced by HMWHA is to promote chondroprotection and involves several proteins including binding to the receptor of the differentiation cluster 44 (CD44), which is required to inhibit the expression of IL-1β, leading to a decline production of matrix metalloproteinases (MMPs) −1, 2, 3, 9, and 13 [7,25,26]. In addition, HA binds hyaluronan-mediated motility receptors (RHAMM) to induce chondroprotection as well as CD44 binding [7]. Regarding the mechanism activated by the binding of CD44, the most important is the role of the mitogen-activated protein kinase phosphatase (MKP)-1, which is able to inhibit the production of IL-1β, and consequently inhibits the MMPs within articular cartilage and finally prevent apoptotic events in the chondrocyte through the reduction in disintegrin and metalloproteinase expression with thrombospondin motifs (ADAMTS) [7,27,28]. Another important element is the production of reactive oxygen species (ROS) and nitric oxide (NO), which are normally involved in the apoptosis-dependent death of chondrocytes, leading to the degeneration of cartilage. In this context, the current literature reported that HMWHA after the binding with CD44 is able to prevent chondrocytes apoptosis by inhibiting PGE2 synthesis and interleukin activity such as IL-1β, which is responsible for oxidative stress [7,25,29,30]. Regarding the effectiveness of HA on joint tissue, it is important to remember that it can act as a passive structural molecule or exerts biological effects via a signaling molecule. Furthermore, since its different mechanism of action depends on the molecular weight, the link between the molecular weight and its pro and anti-inflammatory activities, the promotion, and inhibition of the activation of migration, and the blocking or promotion of the division is also important [10]. Today some details are known about how HA exerts its different biological functions at different concentrations and molecular weights [31]. For example, at the level of the intestinal mucosa, the intermediate HA and HMWHA have antioxidant and antimicrobial properties [32]. Its importance is constantly growing because this substance regulates tissue homeostasis and its physiological decrease is related to the aging process that leads to various diseases [32]. Oral administration of exogenous HA has attracted the attention of researchers as a supplementary therapy to prevent or treat the aging process of cartilage and related diseases [33]. The purpose of this study was to verify the efficacy of HMWHA plant-derived HA (called GreenIuronic^®^) in maintaining joint homeostasis in order to prevent all the harmful processes that can trigger the pathology of OA.

## 2. Results

### 2.1. Characterization of GreenIuronic^®^

**Figure 1:**
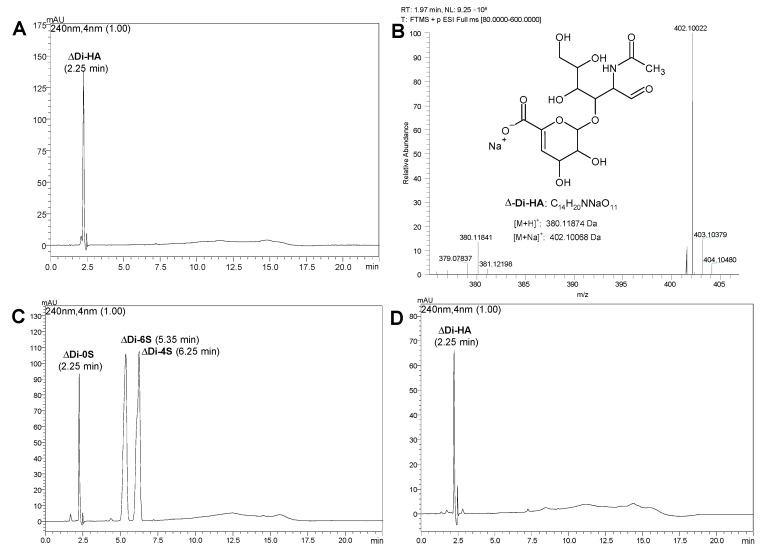
HPLC-UV and high-resolution mass spectrometry (HRMS) analysis of GreenIuronic^®^ after enzymatic hydrolysis with chondroitinase AC. In (**A**,**B**) HPLC-UV chromatogram of GreenIuronic^®^ sample and its positive HRMS spectrum. In (**C**,**D**) HPLC-UV chromatograms of a mixture of chondroitin disaccharides standard ΔDi-0S, ΔDi-4S, and ΔDi-6S and a solution of the disaccharide standard ΔDi-HA of HA.

High-Performance Liquid Chromatography analysis of GreenIuronic^®^ (Figure 1) revealed the presence of HA, which was detected as the corresponding disaccharide ΔDi-HA generated by chondroitinase AC enzymatic hydrolysis of the Tremella extract. The identity of this disaccharide was established by comparison with the ΔDi-HA reference standard and by the protonated and sodiate positive ions detected in its mass spectrum. Moreover, the same analysis also revealed the absence of chondroitin 4 and 6 mono-sulfates, which eluted at 6.25 and 5.35 min, respectively. However, because of the absence of sulfate group in the disaccharide chondroitin 0 sulfate (ΔD-0S), arising from chondroitine hydrolysis, the HPLC method, based on ion-pair retention, did not allow the separation between the disaccharides ΔDi-HA and ΔD-0S: indeed, they eluted at 2.25 min.

Moreover, since HPLC-UV analysis revealed a possible high concentration of HA in GreenIuronic^®^ samples, additional experiments were carried out to quantify the content of glucuronic acid in GreenIuronic^®^ and in sodium hyaluronate samples. As reported in Table 1, the content of glucuronic acid in GreenIuronic^®^ is about 90%, which is higher than that of sodium hyaluronate (about 62%). These data support what was observed in previous experiments in HPLC (reported above) about the purity of the GreenIuronic^®^ material.

**Figure 2:**
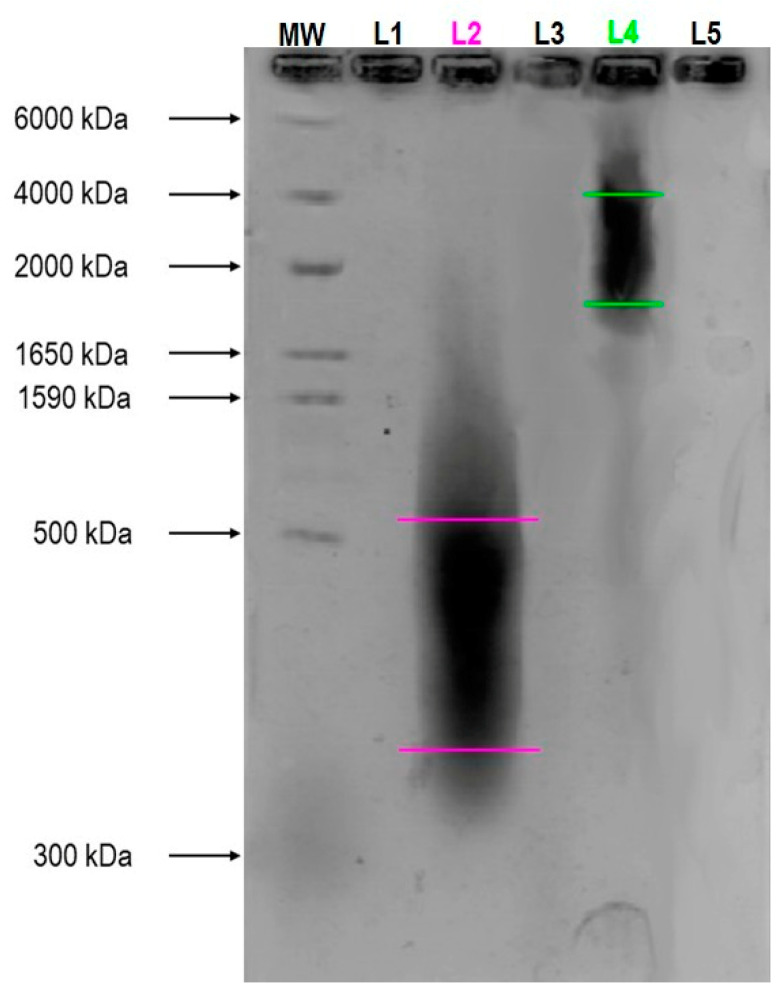
In the figure an example of HA molecular weight determination on 1% Agarose gel. The sample loads are described as follows by the abbreviations: *M*_W_ = standard molecular weight Mega + HiLadder specific for HA detection; L1 = lane empty loaded with 10 µL TAE buffer; L2 = lane loaded with 100 µg/10 µL Sodium Hyaluronate; L3 = lane empty loaded with 10 µL TAE buffer; L4 = lane loaded with 100 µg/10 µL GreenIuronic^®^; L5 = lane empty loaded with 10 µL TAE buffer.

Finally, for the analysis of GreenIuronic^®^ size distribution, agarose gel electrophoresis was used to define a range of the molecular weight. Agarose gel retards the electrophoretic mobility of HA molecules in a molecular weight-dependent manner indicating that GreenIuronic^®^ may be considered the HMWHA (>1650 kDa), as can be seen in Figure 2. On the contrary, sodium hyaluronate was confirmed to have the lower molecular weight HA (LMWHA) (between 300 and 500 kDa). These results indicate that GreenIuronic^®^ molecular weight is higher than that of sodium hyaluronate and further experiments were performed in order to confirm the hypothesis that HMWHA exerts more beneficial effects compared to those of LMWHA.

### 2.2. Dose–Response and Time-Course Study of GreenIuronic^®^ on CaCo-2 Cells

**Figure 3:**
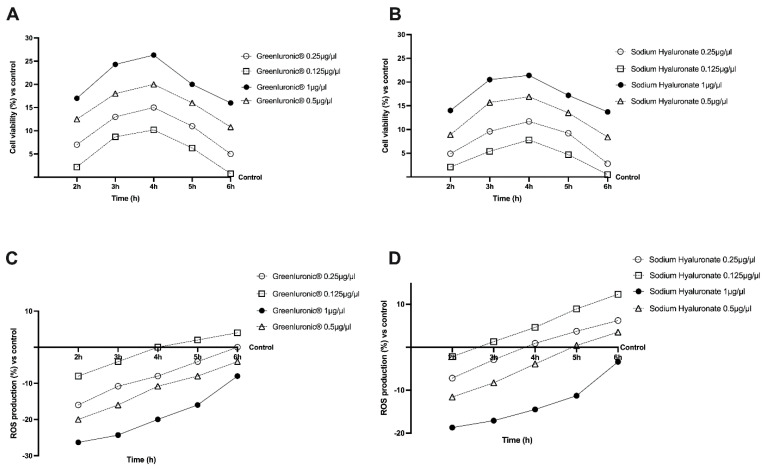
Cell viability and ROS production on CaCo-2 cells. In panel (**A**,**B**) dose–response study on cell viability measured by MTT test of both GreenIuronic^®^ and Sodium Hyaluronate from 2 to 6 h. In panel (**C**,**D**) ROS production of both GreenIuronic^®^ and Sodium Hyaluronate measured by reduction in cytochrome C from 2 to 6 h. Data are mean ± SD of five independent experiments performed in triplicates vs. control values (0% line).

Before studying the permeability and transport of GreenIuronic^®^, the human immortalized colorectal adenocarcinoma (CaCo-2) cell line was used to perform a dose–response study to exclude any cytotoxic effects. The analysis was performed comparing the effects of GreenIuronic^®^ to sodium hyaluronate, testing them at the same concentration (ranging from 0.125 to 1 μg/μL) on cell viability and ROS production in CaCo-2 cells in a time-course study (from 2 to 6 h). The cell viability of the CaCo-2 cells, measured by 3-(4,5-Dimethylthiazol-2-yl)-2,5-diphenyltetrazolium bromide (MTT) assay, showed time and concentration-dependent effects of both substances (Figure 3A), and the beneficial effects compared to control (*p* < 0.05) were maintained during all periods of stimulation excluding any cytotoxic effect at all dosage tested. In particular, the cells treated with GreenIuronic^®^ 1 µg/µL showed high variability compared to control (*p* < 0.05) and compared to other concentrations tested (*p* < 0.05) suggesting that GreenIuronic^®^ 1 µg/µL is non-toxic to intestinal epithelial cells exhibiting the best profile also compared to sodium hyaluronate at the same concentration and time (*p* < 0.05). Additional experiments were carried out in order to confirm the safety of GreenIuronic^®^ on intestinal epithelium analyzing if the substances tested could induce oxidative stress. For this reason, ROS production was evaluated on CaCo-2 cells from 2 to 6 h of stimulations with both GreenIuronic^®^ and sodium hyaluronate. As shown in Figure 3B, none of the concentrations tested was able to increase the ROS production maintaining them at normal physiological conditions. GreenIuronic^®^ 1 µg/µL maintains a low ROS level during all periods analyzed better than the other concentrations tested and all sodium hyaluronate concentrations, and it was maintained for all further experiments.

### 2.3. Permeability Analysis of GreenIuronic^®^ Using an In Vitro Model of Intestinal Barrier

**Figure 4:**
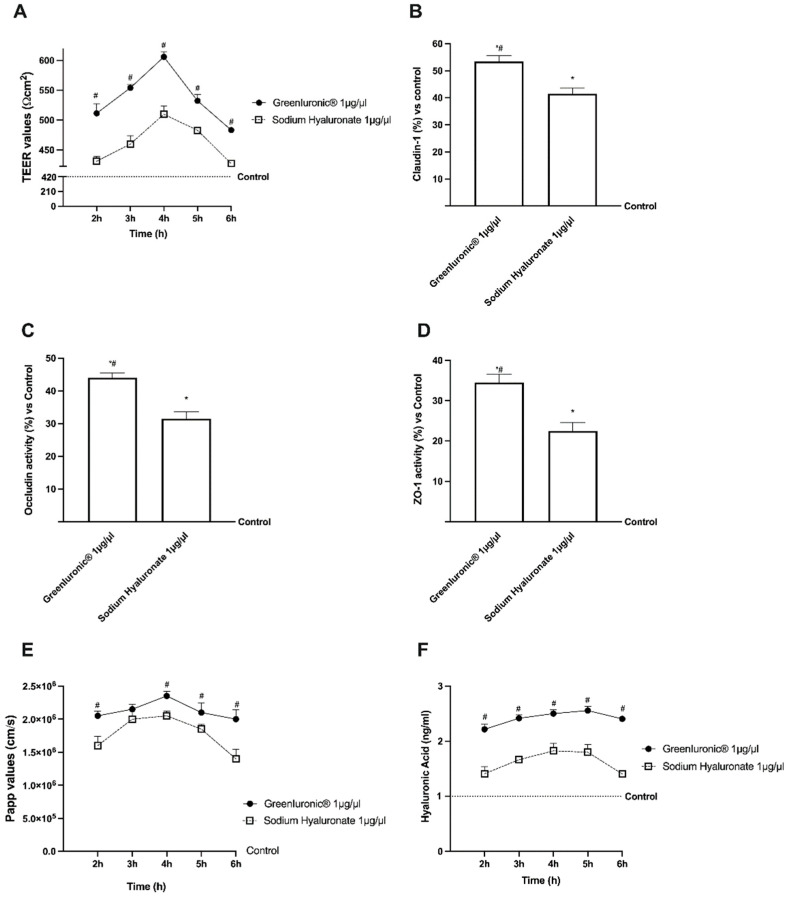
Permeability study on CaCo-2 cells. In (**A**) TEER Value using EVOM3; from (**B**–**D**) the analysis of TJ measured by Enzyme-Linked Immunosorbent Assay (ELISA) test (Occludin, Claudin1, and ZO-1, respectively); in (**E**) the Papp values in which data < 0.2 × 10^−6^ cm/s mean very poor absorption with a bioavailability < 1%, data between 0.2 × 10^−6^ and 2 × 10^−6^ cm/s with bioavailability between 1 and 90%, and data > 2 × 10^−6^ cm/s mean very good absorption with a bioavailability over 90%. In (**F**) HA quantification measured by ELISA kit. Data are mean ± SD of five independent experiments performed in triplicates. From (**B**–**D**) means± SD are expressed comparing data to control value (0% line) and * *p* < 0.05 vs. control; # *p* < 0.05 vs. Sodium Hyaluronate 1 µg/µL. On the contrary, in (**A**,**E**,**F**) the control samples are specifically reported and both GreenIuronic^®^ and sodium hyaluronate are *p* < 0.0001 vs. control; # *p* < 0.05 vs. Sodium Hyaluronate 1 µg/µL.

To assess permeability, and to obtain additional information about the GreenIuronic^®^ intestinal absorption, further experiments were carried out performing a 3D in vitro model in order to mimic the in vivo complexity of the intestinal barrier. In this context, 1 µg/µL GreenIuronic^®^ and 1 µg/µL sodium hyaluronate were tested from 2 to 6 h in order to measure transepithelial electrical resistance (TEER) values, the apparent permeability coefficient (Papp) values, and the HA concentration to predict their bioavailability. The data obtained show that intestinal adsorption has a physiological trend as can be observed from the analysis of TEER and tight junction (TJ). In particular, the passage through the intestinal epithelium demonstrates that both sodium hyaluronate and GreenIuronic^®^ were able to maintain the epithelial integrity increasing the ionic flux of the paracellular exchanges across the intestinal epithelial compared to control (*p* < 0.0001). Indeed, GreenIuronic^®^ demonstrates a better effect compared to sodium hyaluronate during all times of the stimulation (*p* < 0.0001), as reported in Figure 4A. Afterwards, also the evaluation of TJ confirmed these results; indeed, GreenIuronic^®^ exerted the greatest effects on occludin (*p* = 0.0286, about 31%, Figure 4B), claudin-1 (*p* = 0.0299, about 37%, Figure 4C), and zonula occludens-1 (ZO-1) (*p* = 0.0299, about 50%, Figure 4D) compared to sodium hyaluronate and compared to control value (reported as 0 line, *p* < 0.05). From these encouraging results, which confirmed the correct functioning of the intestinal epithelium, further experiments were carried out measuring the permeability rate, analyzing the flux of non-electrolyte tracers (expressed as permeability coefficient as reported) and how much HA has crossed the intestinal barrier to reach the target site. Data obtained from the analysis of the basolateral environment (Figure 4E) confirmed our previous findings since the amount of GreenIuronic^®^ was higher compared to sodium hyaluronate (*p* < 0.05) with a maximum effect at 4 h compared to sodium hyaluronate (about 20%, *p* < 0.013). In addition, the data obtained from the quantification of the basolateral level (Figure 4F) supported the hypothesis about the importance of predicting human absorption; GreenIuronic^®^ has a higher amount of HA that crosses the barrier and reaches the plasma level compared to control (*p* < 0.0001) and compared to sodium hyaluronate (about 30%, *p* < 0.0001) with the greatest effects between 4 and 5 h.

### 2.4. Effects of GreenIuronic^®^ Crossed Intestinal Barrier on Chondrocytes

**Figure 5:**
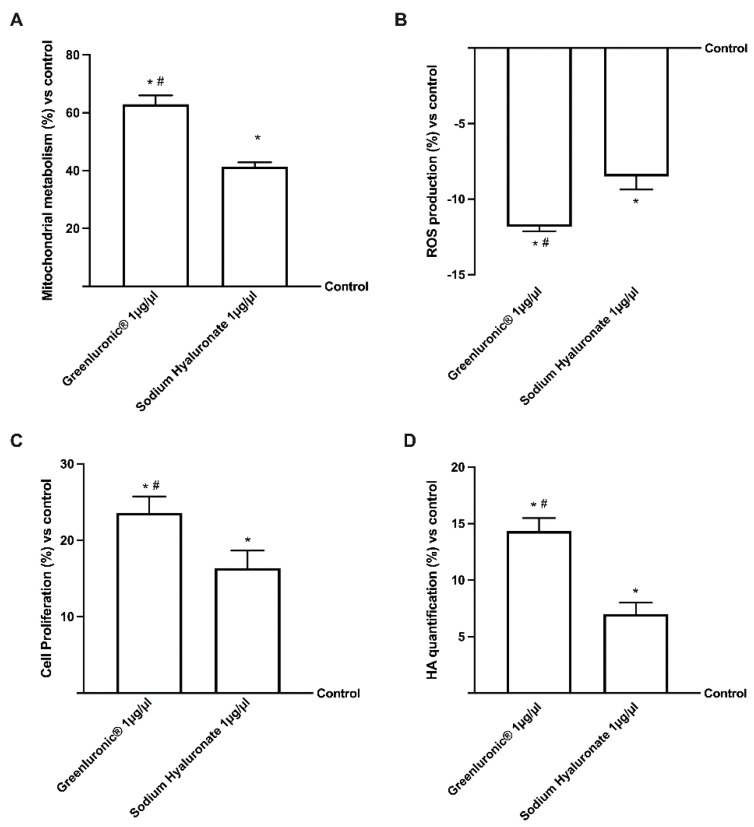
Analysis of GreenIuronic^®^ and Sodium Hyaluronate on human chondrocyte (T/C-28a2) cells functions. In (**A**) the mitochondrial metabolism tested by MTT test; in (**B**) the ROS production; in (**C**) the proliferation analysis by crystal violet assay; and in (**D**) the HA quantification by ELISA kit. Data are expressed as mean ± SD compared to control (0% line) of five independent experiments performed in triplicates. * *p* < 0.05 vs. control; # *p* < 0.05 vs. Sodium Hyaluronate 1 µg/µL.

Since the exogenous hyaluronic acid administered into articular cartilage has a direct biological effect on chondrocytes, several experiments were carried out to explore the effect of GreenIuronic^®^, compared to sodium hyaluronate, on chondrocytes after intestinal absorption in terms of mitochondrial metabolism and cell proliferation. As expected (Figure 5A), both 1 µg/µL GreenIuronic^®^ and sodium hyaluronate were able to improve cell viability compared to control (*p* < 0.05); in particular, GreenIuronic^®^ induces the main effect on cell viability (about 50%, *p* < 0.05) compared to sodium hyaluronate reducing ROS production (about 38% *p* < 0.05), as reported in Figure 5B. Furthermore, as reported in Figure 5C, GreenIuronic^®^ induces an improvement in cell proliferation compared to control (*p* < 0.05), and compared to sodium hyaluronate, by about 60%, indicating that GreenIuronic^®^ is able to stimulate the proliferative activity of chondrocytes. Since the importance of the activity on cell proliferation includes the ability to modulate joint production of HA in cells, the HA quantification in chondrocytes (Figure 5D) revealed that a large amount of HA present in GreenIuronic^®^ and sodium hyaluronate was captured by chondrocytes after intestinal passage compared to the control (*p* < 0.05). In particular, approximately 75% of HA was induced by GreenIuronic^®^ compared to sodium hyaluronate (*p* < 0.05) in chondrocytes, confirming that HMWHA is better utilized by chondrocytes.

### 2.5. Effects of HA Crossed Intestinal Barrier on Chondrocytes under OA Condition

**Figure 6:**
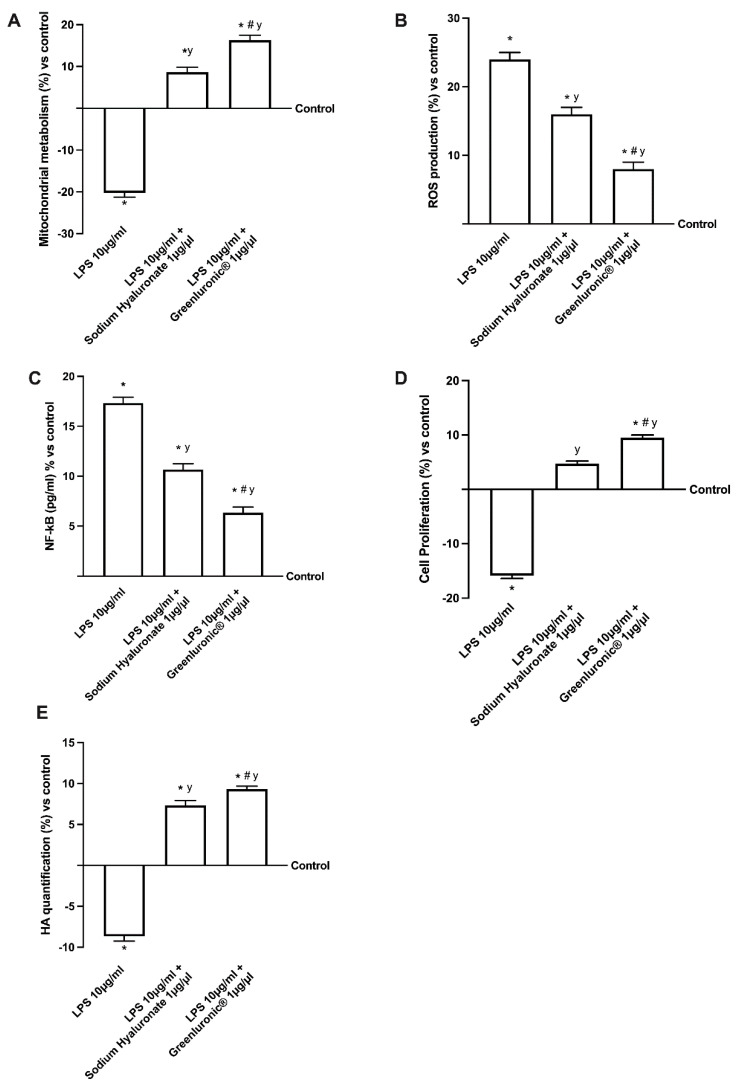
GreenIuronic^®^ and Sodium Hyaluronate effects on T/C-28a2 cells during OA conditions. In (**A**) mitochondrial metabolism tested by MTT test; in (**B**) ROS production; in (**C**) NFkB analysis by ELISA test; in (**D**) proliferation analysis by crystal violet; and in (**E**) HA quantification by ELISA kit. Data are mean ± SD of five independent experiments performed in triplicates expressed as a percentage compared to control (0% line). * *p* < 0.05 vs. control; y *p* < 0.05 vs. 10 µg/mL of LPS; # *p* < 0.05 vs. Sodium Hyaluronate 1 µg/µL.

From the data obtained under physiological conditions, it can be assumed that GreenIuronic^®^ is also effective after oral administration and is an important starting point for determining the success of therapy in joint damage, such as OA. Oxidative stress and inflammation are known to be involved in cartilage degeneration of OA and it is similarly approved that the degree of anti-inflammatory, immunomodulatory, analgesic, and anti-OA effects of HA is determined by *M*_W_ and route of administration. Based on these results, in the last phase, the in vitro study was conducted by analyzing the effects of both 1 µg/µL of GreenIuronic^®^ and sodium hyaluronate on T/C-28a2 cells pretreated with 10 µg/mL of lipopolysaccharide (LPS) for 24 h in order to simulate the condition of OA. The effects of chondrocyte metabolism were shown in Figure 6 where the beneficial effects of GreenIuronic^®^ can be observed. Specifically, chondrocytes treated only with 10 µg/mL of LPS significantly reduced cell viability (panel 6A, about 10%) and improved ROS production (panel 6B about 23%) compared to control (*p* < 0.05) but this effect was significantly reduced by the presence of both agents. In particular, GreenIuronic^®^ was able to counteract these negative effects caused by LPS alone (*p* < 0.05) better than sodium hyaluronate (about 47% on cell viability and two times on ROS production, respectively, *p* < 0.05). These data were also confirmed by nuclear factor kappa B (NFkB) analysis (Figure 6C) in which the beneficial potential of GreenIuronic^®^ against inflammation, a key point in the mechanisms involved during OA processes, was observed. Indeed, the cells treated with only 10 µg/mL of LPS increased inflammatory processes compared to control (about 18%, *p* < 0.05) assuming the beginning of chronic processes leading to cell death, and this situation was reversed following stimulation with both HA agents. In particular, 1 µg/µL GreenIuronic^®^ was able to reduce the negative effect produced by LPS (about 1.25 times more) better than sodium hyaluronate (about 50%, *p* < 0.05). This recovery mechanism was confirmed also by proliferation assay (panel 6D) in which T/C-28a2 cells lost their proliferative properties when treated only with 10 µg/mL of LPS (*p* < 0.05 compared to control). On the contrary, both 1 µg/µL GreenIuronic^®^ and sodium hyaluronate counteract this negative effect compared to control (*p* < 0.05), but GreenIuronic^®^ was able to restore the damage by about 62% compared to sodium hyaluronate (*p* < 0.05), supporting its use during OA injuries. Finally, this recovery mechanism was also confirmed by the analysis of HA, which showed that under OA conditions, GreenIuronic^®^ is able to improve a much higher amount of HA released in stressed chondrocytes than sodium hyaluronate at the same concentration, approximately by about 21% (*p* < 0.05).

**Figure 7:**
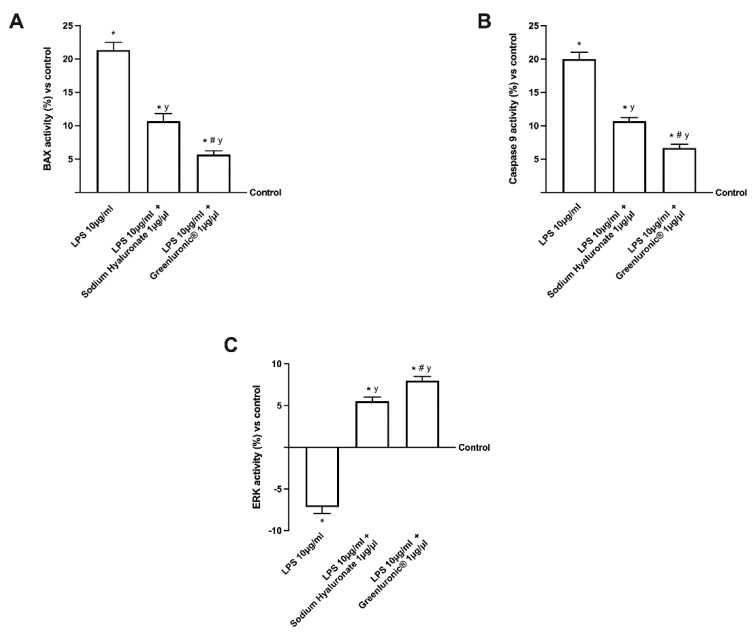
Analysis of the main intracellular pathways activated in T/C-28a2 cells during AO conditions. The results demonstrated a reduction in apoptotic pathways and an improvement of the survival pathways supporting the ability of HA to restore the OA damage. In (**A**) BAX activity; in (**B**) Caspase 9 activity; in (**C**) ERK/MAPK activity; all these results are obtained from specifically ELISA kit. Data are mean ± SD of five independent experiments performed in triplicates compared to the control value (0% line). * *p* < 0.05 vs. control; y *p* < 0.05 vs. 10 µg/mL of LPS; # *p* < 0.05 vs. Sodium Hyaluronate 1 µg/µL.

In order to demonstrate that LPS is able to reproduce the OA condition in vitro leading to chondrocyte death, additional experiments were carried out to explore the involvement of the apoptosis process. As reported in Figure 7, several markers related to apoptotic processes were evaluated in response to 10 µg/mL of LPS and to both 1 µg/mL GreenIuronic^®^ and sodium hyaluronate. In particular, the stimulation with 10 µg/mL of LPS treatment on T/C-28a2 cells enhanced BAX and Caspase 9 activities (Figure 7A,B), about 22% and 18% compared to control (*p* < 0.05), indicating a dramatic improvement of the apoptosis process supporting the chondrocyte death during OA. Contemporary, the stimulation with both 1 µg/µL GreenIuronic^®^ and sodium hyaluronate added after 10 µg/mL of LPS caused a statistically significant reduction in both these markers. In particular, GreenIuronic^®^ exerted the main effects compared to sodium hyaluronate on Bax (about 2 times less, *p* < 0.05) and Caspase-9 activities (about 1.5 times less, *p* < 0.05), suggesting that GreenIuronic^®^ contributes to cell protection. These data were also confirmed by the activation of risk pathways such as mitogen-activated protein kinases/extracellular signal-regulated kinase (ERK/MAPK) activity (Figure 7C), which demonstrated that GreenIuronic^®^ reverts the 10 µg/mL LPS damage, activating the survival pathways and restoring the chondrocyte to normal conditions (about 25% compared to sodium hyaluronate, *p* < 0.05).

**Figure 8:**
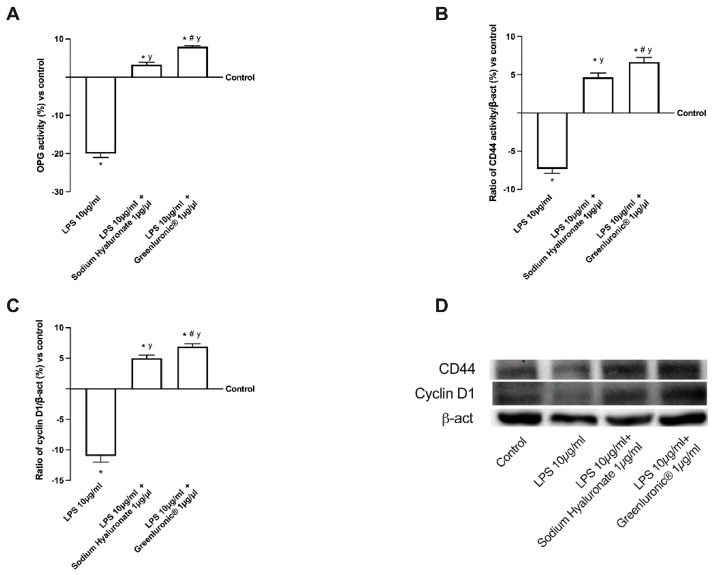
Western-blot and densitometric analysis of the main intracellular pathways activated in T/C-28a2 cells during AO conditions. In (**A**) OPG activity measured by ELISA test, in (**B**) the CD44, and in (**C**) cyclin D1 densitometric analysis of the specific Western blot, which is reported as an example in (**D**). Data are mean ± SD of five independent experiments performed in triplicates compared to control value (0% line). * *p* < 0.05 vs. control; y *p* < 0.05 vs. 10 µg/mL of LPS; # *p* < 0.05 vs. Sodium Hyaluronate 1 µg/µL.

Finally, to explore the possible effector molecules responsible for the maintenance of chondrocyte wellbeing, the activity of cyclin D1, osteoprotegerin (OPG), and CD44 were investigated. As reported in Figure 8A–D, 10 µg/mL of LPS confirmed its negative effect on T/C-28a2 cells compared to control (*p* < 0.05) downregulating OPG activity, CD44 and cyclin D1 expressions (about 18%, 8%, and 12% compared to control, respectively) modifying negatively chondrocytes activity. Conversely, both 1 µg/µL GreenIuronic^®^ and sodium hyaluronate were able to reduce the damage induced by 10 µg/mL of LPS (*p* < 0.05), confirming the positive role of HA contained in two agents in stimulating chondrocyte metabolism. In particular, 1 µg/mL GreenIuronic^®^ appears to be able to induce main effects compared to sodium hyaluronate (*p* < 0.05) to counteract the negative effects of OA induction. Indeed, 1 µg/mL GreenIuronic^®^ is able to restore the damage induced by 10 µg/mL of LPS in all parameters tested (about 60% for OPG, one time more for CD44, and 57% for cyclin D1 expression, *p* < 0.05), suggesting that it could ameliorate chondrocyte pathological conditions by activating them through the markers responsible for articular joint homeostasis.

## 3. Discussion

Current guidelines for the treatment of OA suggest many conventional approaches to improve this chronic condition. For example, pharmacological treatment, which is characterized by NSAIDs [34], opioids, and cyclooxygenase (COX)-2-specific drugs, is an accepted method considered only as a “palliative” method since it reduces the symptoms but does not address the essential problem of cartilage degeneration [35]. In addition, conventional therapies can cause possible side effects, especially for long periods of use, which can reduce the compliance at the onset of gastrointestinal, cardiovascular, and other adverse effects [36]. Furthermore, the conventional therapies often use HA injections to treat knee OA and to improve the functions of the knee joint, these methods are called viscosupplementation [37]. It was reported that intra-articular HA improves synovial fluid elasticity and viscosity by decreasing the release of pain mediators and proinflammatories from synovial cells [37]. Otherwise, a recent systematic review, based on the analysis of pain relief and functional improvement, concluded that the routine use of HA injections does not produce so many benefits for the patient with no clinical relevance, because of the pain caused [38]. According to the current protocol, HA should be administered repeatedly into the joint cavity, but multiple injections could cause much discomfort in patients and increase the risk of complications [39]. Notwithstanding, HA is a useful tool in the management of patients with OA, since clinical data indicate its ability to reduce pain and improve joint function, with the potential ability to modify chondrocytes activity [40]. Also, it should be taken into account that the administration of HA by intraarticular injection can also cause adverse effects such as infectious arthritis and cartilage damage [41]. Therefore, the possibility of administering HA orally represents a considerable advantage [13]. From this point of view, several studies have explored new approaches for consistent and pain-free administration of HA, reporting positive effects after oral administration and suggesting that it may have beneficial therapeutic effects on patients with OA [39,42,43]. Thus, the possibility of using HA in a dietary supplement to be taken orally sparked interest in designing a new nutraceutical (based on plant-derived HA, called GreenIuronic^®^) able to counteract the harmful consequences of OA. With these premises, our chemical analysis revealed that GreenIuronic^®^ contains a large amount of HA with a chemical profile useful to be a new nutraceutical.

In addition, the presence of a high molecular weight ingredient related to HA supports its use to counteract the adverse effects of OA, since high molecular weight HA is nowadays the best treatment option for knee OA by intra-articular injection [44]. However, as reported in the literature, high levels of HA in serum after its oral administration in vivo model are also reported. Indeed, therapeutic efficacy of HA against lameness was found to be greater with oral than intra-articular administration because this way of administration dissipates out of the joint the main amount of HA within 14–18 h; HA diffuses out of tissues via the bloodstream, circulating throughout the body, and is rapidly eliminated [17]. In addition, several human studies have revealed that patients with OA must undergo clinical visits repeatedly and must undergo the discomfort associated with injections while also experiencing an increase in complications associated with repeated injections [39]. Considering these obvious and potential drawbacks, it is much more desirable to use HA by oral administration to improve the condition of OA. Indeed, some studies have suggested that knee OA symptoms may actually be alleviated by taking HA, and other studies also report positive effects of orally administered HA on improving joint function in mild to moderate osteoarthritis of the knee [39,45,46,47]. In addition, the international evidence-based guidelines agree that knee OA management requires both non-pharmacological and pharmacological approaches and suggest initiating a background therapy with chronic symptomatic slow-acting drugs for OA such as HA by oral administration [48]. Consequently, the effects of GreenIuronic^®^ were analyzed mimicking the human oral ministration in vitro, since orally administered HA should be absorbed and distributed to the knee joints where it exerts its biological activities. The results obtained from the 3D model that mimics intestinal absorption clearly demonstrated that oral administration is possible. Bioavailability experiments indicated that orally administered HA is effectively absorbed and biodistributed to the chondrocytes and exerts its biological functions in those tissues. Indeed, GreenIuronic^®^ has a high amount of HA that reaches the plasma level compared to control (*p* < 0.05) and compared to sodium hyaluronate within 4 h and 5 h, confirming the hypothesis that GreenIuronic^®^ improves the absorption during the physiological time of intestinal digestion and improving its bioavailability. In addition, GreenIuronic^®^ treatment indicated that a substantial part of HA is absorbed without damaging the intestinal epithelium; this is a crucial point since HA has a role in decreasing the permeability by enhancing tight junction proteins. In epithelial cells, the formation of tight junctions plays an important role in the intestinal barrier, and this is mediated by proteins such as claudins, occludin, and ZO-1 that are necessary for epithelial barrier activity [49].

These proteins are critical in maintaining homeostatic intestinal permeability in multiple intestinal inflammatory diseases, supporting a gut–joint axis in OA pathogenesis and progression [50]. In particular, dysbiosis-related gut permeability determined lower mRNA levels of TJ, ZO-1, and occludin, and higher LPS plasma levels in the in vivo model, which has a positive association of synovial LPS with inflammation and disease severity in articular chondrocytes in OA patients [51,52]. For this reason, several studies using nutraceuticals evaluate the intestinal integrity as the first step to ameliorate pain and disease progression of OA [50] during chronic nutritional intervention [36], using also HA as a multifunctional agent [9]; in particular, Kotla et al. demonstrated that the treatment with HA upregulated the expression of the tight junction proteins claudin and occludin [53]. These three proteins are pivotal because ZO-1 connects claudin and occludins to the cytoskeleton so they are indicators of good gut barrier functions [54]. Furthermore, it has been demonstrated that GreenIuronic^®^ is able to maintain epithelial integrity and the ionic exchanges across the intestinal barrier, suggesting that this proteoglycan is able to pass the cell monolayer without negatively altering the epithelium. Subsequently, the second important purpose of this work was to test the ability of GreenIuronic^®^ to stimulate chondrocyte biological activity under physiological and pathological conditions. As expected, GreenIuronic^®^ was able to stimulate cell viability and induce chondrocyte proliferation without causing adverse effects, also compared to conventional HA supplementation Indeed, thanks to the presence of HMWHA, the beneficial effects of GreenIuronic^®^ on the activity of chondrocytes support the hypothesis of its use in inflammatory joint conditions. Since OA is a disease of the whole joint and a multifactorial entity, there are various therapeutic strategies that involve numerous fields of medicine: rheumatology, orthopedics, geriatrics, psychiatry, general practitioners, and physiotherapists. The goal of OA therapy is to reduce pain and increase patients’ quality of life. For this purpose, HA has shown not only beneficial effects on articular cartilage trophism, but also antinociceptive effects with a significant reduction in pain [55]. In particular, the beneficial effects of GreenIuronic^®^ have also been confirmed by the quantity of HA, contained in this new formulation, which reached the target site and was absorbed into the joint without damaging it. Consequently, the therapeutic effects of GreenIuronic^®^ on OA conditions may necessarily require the improved absorption of HA. Moreover, we pre-treated T/C-28a2 cells with 10 µg/mL of LPS to mimic the osteoarthritic phenotype as reported by Zhang et al. [56]. Our data support the literature [57,58] showing that HMWHA may bind to CD44 on chondrocytes to exert its biological activities, demonstrating that the association of HA with CD44 increased the HA absorption/production suppressing proinflammatory processes. The binding of HA, present in GreenIuronic^®^, to CD44 also suppresses the expression of the apoptosis process, which again contributes to the damage of chondrocytes and improves its activity, regulating cartilage production, improving OPG activity, and proliferation process, modulating cyclin D1 expression. Taken together, these results suggest that GreenIuronic^®^ is the best choice to maintain chondrocyte behavior during the inflammatory condition related to OA, and therefore, the application of this innovative HA form could be an excellent strategy to restore OA damage.

## 4. Materials and Methods

### 4.1. Agents Preparation

GreenIuronic^®^ was obtained from White Tremella (Silver Ear), which is a traditional foodstuff with medicinal applications in China [59]. The production process involves several steps necessary to obtain a final extract and includes a new technology based on patent N°WO2021/250566 from Vivatis Pharma GBHE, Grüner Deich 1–3, 20,097 Hamburg, Germany. Briefly, the process involves steps of extraction, purification, and refining by alcohol solution, sieving, and crushing. The resulting powder is then packed and tested for metals and stored [60,61]. In addition, sodium hyaluronate (Merck Life Science, Rome, Italy) was tested to verify the mechanism of action of GreenIuronic^®^. All these substances are prepared directly in water for HA determination or directly in Dulbecco’s Modified Eagle’s Medium (DMEM, Merck Life Science, Rome, Italy) without phenol red and supplemented with 0.5% fetal bovine serum (FBS, Merck Life Science, Rome, Italy), 2 mM L-glutamine (Merck Life Science, Rome, Italy), and 1% penicillin–streptomycin (Merck Life Science, Rome, Italy) for biological analysis.

### 4.2. HPLC Analysis

The determination of the HA was also confirmed by HPLC (Shimadzu, Kyoto, Japan) analysis according to the method reported in the literature [62], as described in Appendix A (Appendix A.1 and Appendix A.2). Briefly, 20 μL of TRIS buffer (3.0 g TRIZMA base, 4.0 g sodium acetate trihydrate, 1.46 g sodium chloride, and 50 mg crystalline bovine serum albumin dissolved in 100 mL of 0.12 M HCl, pH 7.3 with 6 M HCl. All chemicals are purchased from Merck Life Science, Rome, Italy), 30 μL of chondroitinase AC solution (Merck Life Science, Rome, Italy) (diluted to 10 U/mL with water), and 20 μL of GreenIuronic^®^ test solution (200 mg dissolved in 100 mL of water) were pipetted into a conical 1.5 mL vial. The vial was placed in a warm water bath at 37 °C for 3 h. After cooling at room temperature, the sample was diluted to 1 mL by adding 930 μL of mobile phase A (reagent purchased from Merck Life Science, Rome, Italy, and column from Phenomenex Srl, Bologna, Italy) (see Appendix A.1 and Appendix A.2 in Appendix A) and the mixture was analyzed by HPLC-UV and HPLC-HRMS systems. A control solution was prepared by replacing the enzyme aliquot with TRIS buffer.

### 4.3. Colorimetric Determination of Hyaluronic Acid

The assay performed to quantify the concentration of HA on material samples was the same reported in the literature [63]. Briefly, 1 mg of raw sample was dissolved in 1 mL of deionized water, and 200 µL of resuspended samples were displaced in new Eppendorf, diluted with 1.2 mL of sulfuric acid (Merck Life Science, Rome, Italy) with 0.0125 M tetraborate (Merck Life Science, Rome, Italy), shaken for 20 s and then boiled at 100 °C for 5 min. Once the samples were allowed to cool on ice, 20 µL of 0.15% hydroxydiphenyl (Merck Life Science, Rome, Italy) (dissolved in 0.5% NaOH, Merck Life Science, Rome, Italy) was added and stirred; 100 µL of each sample was placed in a 96 multi-well plate and the absorbance was measured at 340 nm by a spectrophotometer (Infinite 200 Pro MPlex, Tecan, Männedorf, Switzerland). The data obtained were compared to a calibration curve generated using glucuronic acid (0, 0.25, 0.5, 1, 1.5, 2 mg/mL Merck Life Science, Rome, Italy) [64] and the results were expressed as mean (%*w/w*) ± SD compared to control (0 line).

### 4.4. Molecular Weight Determination

The determination of the molecular weight of HA before exploring its biological effects was carried out using 1% agarose gel, following the method reported in the literature [65]. Briefly, 0.3 g agarose (Merck Life Science, Rome, Italy) was dissolved in 30 mL of Tris-acetate-EDTA (TAE) buffer (48.5 g tris base, 11.4 mL acetic acid, and 0.5 M EDTA pH 8, all substances were purchased from Merck Life Science, Rome, Italy) and the solution was heated for 30 s in a microwave at high power. The gel was poured into the holder and allowed to solidify before performing a pre-run at 100 V for 45 min, using the Mini-Sub Cell GT System (Bio-Rad, Hercules, CA, USA). In the meantime, samples were prepared by dissolving 200 µg of raw samples in 16 µL of TAE buffer 1×. Before running the gel, 4 µL of loading buffer (0.2% Bromophenol Blue, 1 mL of TAE 1×, and 8.5 mL of glycerol, which were purchased from Merck Life Science, Rome, Italy) was added to each sample and to the molecular weights (mixture of 5 µL of Select-HA HiLadder and 5 µL Select-HA Mega Ladder, Echelon Biosciences, Tebu-Bio Srl, Magenta, Italy). The samples were run at 100 V until the samples reached 1 cm from the end of the gel. Then, the gel was hydrated in H_2_O for 24 h at room temperature in agitation and then the gel was placed in 30% ethanol with 0.015% Stains All dye (Merck Life Science, Rome, Italy) for 24 h in the dark. The gel was decolored for 30 min in H_2_O in the dark before proceeding with image acquisition using ChemiDoc™ Touch Imaging System (Bio-Rad, Hercules, CA, USA). The image obtained was analyzed by Image Lab 3.0 software (Bio-Rad Hercules, CA, USA).

### 4.5. Cell Culture

The human epithelial intestinal cells, CaCo-2, purchased from the American Type Culture Collection (ATCC), were cultured in Dulbecco’s Modified Eagle’s Medium/Nutrient F-12 Ham (DMEM-F12, Merck Life Science, Rome, Italy) containing 10% FBS (Merck Life Science, Rome, Italy), 2 mM L-glutamine and 1% penicillin–streptomycin (Merck Life Science, Rome, Italy) maintaining in an incubator at 37 °C and 5% CO_2_ [66]. The cells used for the experiments were at passage numbers between 26 and 32 in order to preserve the integrative paracellular permeability and transport properties [67] maintaining the similarity to the intestinal absorption mechanism following oral intake in humans. The cells were plated in a different manner to perform several experiments including 1 × 10^4^ cells in 96 well plates to study cell viability by MTT-based In Vitro Toxicology Assay Kit (Merck Life Science, Rome, Italy) and ROS production using cytochrome C (Merck Life Science, Rome, Italy) in a complete medium. Eight hours before the stimulation the cells were incubated with DMEM without red phenol and supplemented with 0.5% FBS (Merck Life Science, Rome, Italy), 2 mM L-glutamine, and 1% penicillin–streptomycin (both from Merck Life Science, Rome, Italy) at 37 °C to synchronize them. In addition, 2 × 10^4^ cells were plated on 6.5 mm Transwell^®^ (Corning^®^ Costar^®^, Merck Life Science, Rome, Italy) with a 0.4 μm pore polycarbonate membrane insert (Corning^®^ Costar^®^, Merck Life Science, Rome, Italy) in a 24 well plate to perform the absorption analyses [68]. Cells plated on the Transwell^®^ insert were maintained in a complete medium, which was changed every other day on the basolateral and apical sides for 21 days before the simulations [69]. Before the stimulation, on the apical side, the medium was brought to pH 6.5 as the pH in the lumen of the small intestine, while the pH 7.4 on the basolateral side represented blood [70,71]. This in vitro model is widely used [68,72] and accepted by European Medicines Agency (EMA) and FDA to predict the absorption, metabolism, and bioavailability of several substances after oral intake in humans [73,74].

The immortalized human juvenile costal chondrocyte cell line T/C-28a2 (purchased from Merck Life Science, Rome, Italy) was cultured in DMEM-F12 medium supplemented with 10% FBS (Merck Life Science, Rome, Italy), 2 mM L-glutamine (Merck Life Science, Rome, Italy), and antibiotics (50 UI/mL penicillin and 50 μg/mL streptomycin, Merck Life Science, Rome, Italy)) and maintained in an incubator at 5% CO_2_ and 95% humidity [75]. This cell line is representative and the most commonly used cells for mimicking joints [76] and they were used between passages 3 and 10 [77]. For the experiments 1 × 10^4^ cells were seeded in 96 well plates to study cell viability by MTT-based In Vitro Toxicology Assay Kit (Merck Life Science, Rome, Italy), ROS production using cytochrome C (Merck Life Science, Rome, Italy), and Crystal Violet (Merck Life Science, Rome, Italy) in a complete medium; additionally, 1 × 10^6^ cells were plated on a 6-well to determine HA concentration, using quantification kit, and to analyze molecular pathways by Western-blot analysis or ELISA kit.

### 4.6. Experimental Protocol

In order to analyze the beneficial effects of hyaluronic acid on articular joints in humans after oral intake, the experiments were divided into two steps; the aim of the first one was to verify the ability of HA to cross the intestinal barrier in vitro model excluding negative effects, and of the second one was to check the direct effects on chondrocytes analyzing several parameters and mechanism of actions. For this reason, in intestinal CaCo-2 cells, a dose–response study ranging from 0.125 to 1 μg/μL [78] was performed to assess the concentration able to exert beneficial effects on cell viability and ROS production. Subsequently, the best concentration of GreenIuronic^®^ and hyaluronic acid salt were tested on an intestinal in vitro barrier model to verify intestinal integrity through TEER measurement, tight junction analysis by ELISA kit, and permeability assay by Papp measurement, also analyzing the total amount of hyaluronic acid that had crossed the intestinal barrier. For all these experiments, cells were treated in a time-dependent manner from 2 to 6 h, as reported in the literature [66]. In addition, after each stimulation, the basolateral medium was collected to be used on chondrocytes cells. T/C-28a2, a chondrocyte cell line widely used to study articular joints, was treated for 3 days [79] and, at the end of stimulation, the mitochondrial metabolism, cell proliferation, ROS production, and hyaluronic acid quantification were tested. Finally, in order to mimic OA conditions, further experiments were performed pre-treating T/C-28a2 with 10 µg/mL of LPS (Merck Life Science, Rome, Italy) for 24 h [80], and then stimulating with GreenIuronic^®^ and sodium hyaluronate for 3 days to evaluate if they are able to restore the damage. In these conditions, the survival mechanisms and articular recovery were investigated.

### 4.7. Cell Viability

The analysis of cell viability was performed using a classical technique based on the MTT-based In Vitro Toxicology Assay Kit (Merck Life Science, Rome, Italy) [81], following the manufacturer’s instructions. Indeed, at the end of stimulation, the cells were incubated with 1% MTT dye for 2 h in an incubator at 37 °C, 5% CO_2_, and 95% humidity, and then the purple formazan crystals were dissolved in an equal volume of MTT Solubilization Solution. The absorbance was analyzed by spectrophotometer (Infinite 200 Pro MPlex, Tecan, Männedorf, Switzerland) at 570 nm with correction at 690 nm, and results were expressed compared to the control (0% line), which represented untreated cells. The results reported an increase in the percentage of viable cells compared to the control and indicated a higher number of viable cells plus the control. This strategy can lead to a high level of safety of the stimulation and, consequently, to a correct analysis of the results.

### 4.8. In Vitro Intestinal Barrier Model

An intestinal barrier model, using CaCo-2 cells, was performed to analyze the passage through the intestinal barrier of GreenIuronic^®^ and sodium hyaluronate, having, as a final destination, the chondrocyte where they could exert their beneficial effects. For this reason, the TEER values were determined with EVOM3, coupled with STX2 chopstick electrodes (World Precision Instruments, Sarasota, FL, USA); this assay was carried out every 2 days for 21 days until reaching a TEER value ≥ 400 Ωcm^2^ before the stimulation [66,82], the time required for the cell monolayer formation, for cell differentiation, and for the exposition of the intestinal villi. On day 21, the medium at the apical and basolateral environments was changed to create different pH conditions: pH around 6.5 at the apical level (acidic pH mimicking lumen of small intestine) and pH around 7.4 at the basolateral level (neutral pH mimicking human blood) [69]. The cells were kept for 15 min at 37 °C and 5% CO_2_, after that, the TEER values were measured again before the start of the experiment to verify the stabilization of the values. The cells were stimulated with GreenIuronic^®^ and sodium hyaluronate for 2 h to 6 h before the successive analysis, including the permeability assay measured by Papp analysis [66]. Briefly, the Papp (cm/s) was calculated with the following formula [66,69]:

Papp = dQ/dt ⇥ 1/m0 ⇥ 1/A ⇥ V Donor

dQ: amount of substance transported (nmol or μg);

dt: incubation time (sec);

m0: amount of substrate applied to donor compartment (nmol or μg);

A: surface area of Transwell membrane (cm^2^);

VDonor: volume of the donor compartment (cm^3^).

Negative controls without cells were tested to exclude Transwell membrane influence.

### 4.9. Occludin Quantification Assay

The Human Occludin ELISA kit (OCLN kit, MyBiosource, San Diego, CA, USA) analyzed the occludin presence in CaCo-2 cell lysates, according to the manufacturer’s instruction [66]. Briefly, CaCo-2 cells were lysed with cold Phosphate-Buffered Saline (PBS, Merck Life Science, Rome, Italy) 1×, centrifuged at 1500× *g* for 10 min at 4 °C, and 100 μL of each sample was transferred to the strip well before the incubation at 37 °C for 90 min. The supernatants were removed, and the strips were incubated with 100 μL of Detection Solution A for 45 min at 37 °C; then, the strips were washed with Wash Solution and incubated with 100 μL of Detection Solution B for an additional 45 min. At the end of this time, 90 μL of Substrate Solution was added followed by an incubation for 20 min at 37 °C in the dark, and then 50 μL of Stop Solution was used to block the enzymatic reaction. The plate was analyzed by a spectrophotometer (Infinite 200 Pro MPlex, Tecan, Männedorf, Switzerland) at 450 nm. The concentration is expressed as pg/mL compared to a standard curve (range from 0 to 1500 pg/mL) and the results are expressed as percentage (%) versus control (0 line).

### 4.10. Claudin 1 Detection

The Human Claudin1 was measured in CaCo-2 lysates by ELISA kit (Cusabio Technology LLC, Huston, TX, USA), following the manufacturer’s instructions [66]. Briefly, the cells were lysed with cold PBS 1× (Merck Life Science, Rome, Italy) and centrifuged at 1500× *g* for 10 min at 4 °C. Then, 100 μL of each sample was added to the ELISA plate and incubated at 37 °C for 2 h; after which, the plate was washed and 100 μL of Biotin-antibody was added to the wells and incubated for 1 h at 37 °C. After this time, the wells were washed and 100 μL of HRP-avidin were added in each well, and the samples were incubated for 1 h at 37 °C. Then, 90 μL of TMB Substrate was also added to the samples and the plate was incubated for 20 min at 37 °C protected from light. At the end, 50 μL of Stop Solution was used to stop the reaction and the plate was analyzed by a spectrophotometer (Infinite 200 Pro MPlex, Tecan, Männedorf, Switzerland) at 450 nm. The concentration was expressed as pg/mL, comparing data to the standard curve (range from 0 to 1000 pg/mL), and the results were expressed as percentage (%) versus control (0 line).

### 4.11. ZO-1 Detection

The Human Tight Junction Protein 1 ELISA kit (MyBiosource, San Diego, CA, USA) was measured in CaCo-2, following the manufacturer’s instructions [66]. Briefly, the cells were rinsed with ice-cold PBS 1× (Merck Life Science, Rome, Italy) and processed with two freeze-thaw cycles; then, cell lysates were centrifuged for 5 min at 5000× *g* at 4 °C. After which, 100 µL of each sample were collected and incubated on the ELISA plate at 37 °C for 90 min; after washing, 100 μL of Detection Solution A was added to each well and incubated for 45 min at 37 °C. The wells were washed and 100 μL Detection Solution B was added to the samples. After an incubation of 45 min, the wells were washed again and 90 μL of Substrate Solution was added to each well, and then the samples were incubated for 20 min at 37 °C in the dark. Finally, 50 μL of Stop Solution was added and then the plates were read by a spectrophotometer (Infinite 200 Pro MPlex, Tecan, Männedorf, Switzerland) at 450 nm. The concentration was expressed as pg/mL, comparing data to standard curve (range from 0 to 1000 pg/mL), and the results were expressed as percentage (%) versus control (0 line).

### 4.12. Crystal Violet Staining

At the end of stimulation time, the cells were fixed with 1% glutaraldehyde (Merck Life Science, Rome, Italy) for 15 min at room temperature, washed, and stained with 100 µL 0.1% aqueous crystal violet (Merck Life Science, Rome, Italy) for 20 min at room temperature and solubilized with 100 µL 10% acetic acid before reading the absorbance at 595 nm using a spectrophotometer (Infinite 200 Pro MPlex, Tecan, Männedorf, Switzerland). The estimated number was determined by comparing data to the control cells normalized to T0 (measurement at the beginning of the stimulation) [83]. The results were expressed as percentage (%) versus control (0 line).

### 4.13. ROS Production

The quantification of superoxide anion release was obtained following a standard protocol based on the reduction in cytochrome C [83], and the absorbance in culture supernatants was measured at 550 nm using the spectrophotometer (Infinite 200 Pro MPlex, Tecan, Männedorf, Switzerland). The O_2_ rate was expressed as the mean ± SD (%) of nanomoles per reduced cytochrome C per microgram of protein compared to the control (0 line) [83].

### 4.14. Quantification of Hyaluronic Acid in Cell Culture

At the end of stimulations, both cell types were lysed with 100 µL of cold PBS1× to measure the total HA following the instructions of the Hyaluronic Acid ELISA Kit (ClueClone). Briefly, 50 µL of sample and reagent A were added to each well and after gently shaking the plate was incubated for 1 h at 37 °C. At the end, the wells were washed three times and 100 µL of reagent B was added before incubating the plate for 30 min at 37 °C, then 90 µL of substrate solution was added before incubating the plate for 20 min at 37 °C. At the end, 50 µL of stop solution was added immediately before reading at 450 nm by a spectrophotometer (Infinite 200 Pro MPlex, Tecan, Männedorf, Switzerland) [84,85]. The results were expressed as means ± SD (%) versus control (0 line).

### 4.15. ERK/MAPK Activity

The analysis of ERK/MAPK activity was performed using the InstantOneTM ELISA (Thermo Fisher, Milan, Italy) on chondrocytes lysates [86]. Briefly, 50 μL of lysate samples prepared in Lysis Buffer were tested in ELISA microplate strips after the incubation for 1 h at room temperature in a microplate shaker pre-coated with the antibody cocktail. After that, the strips were incubated with the detection reagent for 20 min before stopping the reaction with a stop solution. The absorbance was measured by a spectrophotometer at 450 nm (Infinite 200 Pro MPlex, Tecan, Männedorf, Switzerland) and the results were expressed as means ± SD (%) versus control (0 line).

### 4.16. OPG Activity

The OPG/TNFRSF11B Duo Set (R&D Systems, Minneapolis, MN, USA) was applied according to the manufacturer’s instructions to verify the OPG involvement [87]. Briefly, 100 μL of samples or standards were added to the well and incubated for 2 h at room temperature protected from light and, after washing, 100 μL of the Detection Antibody was added to each well and incubated as previously described. After 2 h, 100 μL of the working dilution of Streptavidin-HRP A was added to each well and incubated for 20 min at room temperature. At the end of the time, 100 μL of Substrate Solution was added to each well, incubated for 20 min at room temperature, and then 50 μL of Stop Solution was used to stop the enzymatic reaction. The absorbance of each well was measured at 450 nm by a spectrophotometer (Infinite 200 Pro MPlex, Tecan, Männedorf, Switzerland) and the results were interpolated with the standard curve (6.25 to 625 pg/mL) and the results were expressed as means ± SD (%) compared to control (0 line).

### 4.17. NFKB Analysis

The NF-kB (p65) Transcriptional factor Assay kit was carried out to analyze the NF-κB DNA binding activity, following the manufacturer’s instruction (Cayman Chemical Company, Ann Arbor, MI, USA) [88]. The concentration was calculated by comparing results to the standard curve (generated by NF-kB (p65) Transcriptional factor positive control (ranging from 0 to 10 µL/well according to differently scaled dilutions) and reported as means ± SD (%) compared to control (0 line).

### 4.18. BAX Assay

BAX activity was determined in chondrocyte lysates using an ELISA kit (Human Bax ELISA Kit, MyBiosource, San Diego, CA, USA) according to the manufacturer’s instructions [89]. The absorbance of the samples was measured at 450 nm by a spectrophotometer (Infinite 200 Pro MPlex, Tecan, Männedorf, Switzerland) and the results were compared to the standard curve (range from 0 to 2000 pg/mL) and expressed as means ± SD (%) normalized to control value (0 line).

### 4.19. Caspase 9 Assay

The Caspase 9 activity was investigated in chondrocytes lysates by ELISA kit (Caspase 9 Human ELISA Kit, Thermoscientific, Waltham, MA, USA), according to the manufacturer’s instructions, reading the sample’s absorbance at 450 nm with a spectrometer (Infinite 200 Pro MPlex, Tecan, Männedorf, Switzerland). The data were obtained by comparison to a standard curve (ranging from 1.6 to 100 ng/mL), and the results were expressed as means ± SD (%) compared to control value (0 line) [90].

### 4.20. Western-Blot Analysis

At the end of each stimulation, chondrocytes were washed with ice-cold PBS 1× (Merck Life Science, Rome, Italy), and lysed using Complete Tablet Buffer (Roche, Basel, Switzerland) supplemented with 2 mM sodium orthovanadate (Na_3_VO_4_), 1 mM phenylmethanesulfonyl fluoride (PMSF) (Merck Life Science, Rome, Italy), 1:50 mix Phosphatase Inhibitor Cocktail (Merck Life Science, Rome, Italy), and 1:200 mix Protease Inhibitor Cocktail (Merck Life Science, Rome, Italy) to obtain a total protein extract that was centrifuged at 14,000× *g* for 20 min at 4 °C. Then, 35 µg of proteins for each extract was resolved on 8% and 10% SDS-PAGE gel and transferred to a polyvinylidene difluoride (PVDF) membrane, which was incubated overnight with the specific primary antibodies such as Cyclin D1 (1:500, Santa Cruz, CA, USA) and CD44 (1:500, Santa Cruz, CA, USA). All protein expressions were normalized and verified through β-actin detection (1:5000, Merck Life Science, Rome, Italy), and expressed as mean ± SD (%) compared to control value (0 line).

### 4.21. Statistical Analysis

Data obtained from each experimental protocol and assay were collected and analyzed using GraphPad Prism 7 statistical software through mixed variance analysis. In particular, for all growth curves, bar graphs, and line graphs, five independent experiments were performed in triplicates and included in the statistical analysis. All time points in growth curves were presented as the mean of the three biological replicates with mean errors < 5%. The two-tailed Student’s *t*-test was followed by Welch’s t test to analyze two groups. Multiple comparisons between groups were analyzed by two-way ANOVA followed by a two-tailed Dunnett post hoc test. Error bars in the bar charts and line charts represent the standard deviation. For TEER analyses, one-way ANOVA followed by Bonferroni post hoc tests was performed to see if the means were significantly different between groups. All results were expressed as mean ± SD of at least 5 independent experiments performed in triplicates. Differences with a *p* value < 0.05 were considered statistically significant. Data normality was assessed with the Kolmogorov–Smirnov test.

## 5. Conclusions

As demonstrated by these findings, the results of our study show that this new form of plant HA is likely absorbed and distributed to the chondrocytes, while preserving its biological activities. Although the in vitro data are very clear and promising, in vivo or even human studies would be needed to confirm these observations, before assuming an absolute efficacy of this HA extracted from plants. Thus, despite the fact that our data derived from an in vitro study and, therefore, need further validation, the results of the present study about the effectiveness in improving chondrocyte function in conditions that mimic OA, may support the hypothesis that the oral administration of GreenIuronic^®^ in humans can be considered a valid therapeutic strategy to obtain beneficial therapeutic effects during OA. In particular, it can be hypothesized that these promising beneficial effects are relevant not only to the joint, but also to any OA-induced damage.

## Figures and Tables

**Table 1:** Quantification of HA. The % *w/w* of all HA forms normalized on standard curves generated using glucuronic acid standard (ranging from 0 to 2 mg/mL) analyzed at 340 nm by spectrophotometry (Infinite 200 Pro MPlex, Tecan). Data are expressed as means ± standard deviation (SD) (%) of five independent experiments performed in triplicates.

Raw Material	Mean (%*w/w*) ± SD
Sodium Hyaluronate	62.5 ± 2.121
GreenIuronic^®^	90.5 ± 6.364

## Data Availability

Raw data are preferably deposited at the Laboratory of Physiology (C. Molinari), ensuring appropriate measures so that raw data are retained in full forever under a secure system. The data presented in this study are available on reasonable request from the corresponding author.

## Abbreviations

ADAMTS	disintegrin and metalloproteinase with thrombospondin motifs
ANOVA	one-way analysis of variance
CaCo-2	the human immortalized colorectal adenocarcinoma cell line
CD44	differentiation cluster 44
COX-2	cyclooxygenase 2
DMEM/F12	Dulbecco’s modified Eagle’s medium/Nutrient F-12 Ham
EFSA	European Food Safety Authority
ELISA	Enzyme-Linked Immunosorbent Assay
EMA	European Medicines Agency
ERK	extracellular signal-regulated kinases
ERK/MAPK	mitogen-activated protein kinases/extracellular signal-regulated kinase
FBS	fetal bovine serum
FBS	fetal bovine serum
FDA	US Food and Drug Administration
GAGs	glycosaminoglycan heteropolysaccharides family
HA	hyaluronic acid
HMWHA	high-molecular weight HA
HPLC	High-Performance Liquid Chromatography
HRMS	high-resolution mass spectrometry
IBD	inflammatory bowel disease
IL-1β	interleukin (IL)-1β
LMWHA	low molecular weight HA
LPS	lipopolysaccharide
MTT	3-(4,5-Dimethylthiazol-2-yl)-2,5-diphenyltetrazolium bromide
MMPs	matrix metalloproteinases
MPK-1	mitogen-activated protein kinase phosphatase-1
MTT	3-(4,5-Dimethylthiazol-2-yl)-2,5-Diphenyltetrazolium Bromide
Na3VO4	sodium orthovanadate
NFkB	nuclear factor kappa B
NSAIDs	non-steroidal anti-inflammatory drugs
NO	nitric oxide (NO)
OA	Osteoarthritis
OPG	osteoprotegerin
Papp	apparent permeability coefficient
PBS	phosphate-buffered saline
PGE2	prostaglandin E2
PMSF	phenylmethanesulfonyl fluoride
PVDF	polyvinylidene difluoride
RHAMM	hyaluronan-mediated motility receptors
ROS	reactive oxygen species
TEER	transepithelial electrical resistance
T/C-28a2	human chondrocyte cells
TJ	tight junction
ZO-1	zonula occludens-1

## Appendix A

### Appendix A.1. HPLC-UV Method

- Column: Phenomenex Synergi Polar 4 µm 150 × 4.6 mm preceded by a Security guard Polar and kept at room temperature
- Mobile phase A: 340 mg of tetrabutylammonium bisulfate dissolved in 1000 mL of water HPLC grade.
- Mobile phase B: 340 mg of tetrabutylammonium bisulfate dissolved in 330 mL of water HPLC grade, then after the solution is at room temperature, brought to 1000 mL with acetonitrile.
- Wavelength: 240 nm
- Volume of injection: 30 µL
- Flow rate: 1.1 mL/min
- Gradient elution program:
  **Time (min)**	**Mobile Phase B%**
  0.00	20
  7.00	65
  12.00	65
  12.50	20
  22.50	20

### Appendix A.2. HPLC-HRMS Method

- Thermo Scientific Q-Exactive plus
- Column: Phenomenex Synergi Polar 4 µm 150 × 2.0 mm preceded by a Security guard Polar and kept at room temperature
- Mobile phase A: 0.1% formic acid in water
- Mobile phase B: 0.1% formic acid in acetonitrile
- Volume of injection: 5 µL
- Flow rate: 0.200 mL/min
- Gradient elution program:
  **Time (min)**	**Mobile Phase B%**
  0.00	15
  4.00	50
  9.50	50
  10.00	15
  15	15
- Positive full scan.
